# Acute Prosthetic Joint Infections with Poor Outcome Caused by Staphylococcus Aureus Strains Producing the Panton–Valentine Leukocidin

**DOI:** 10.3390/biomedicines11061767

**Published:** 2023-06-20

**Authors:** Martina Maritati, Marco Manfrini, Maria Rosa Iaquinta, Alessandro Trentini, Silva Seraceni, Matteo Guarino, Anna Costanzini, Roberto De Giorgio, Gustavo Alberto Zanoli, Alessandro Borghi, Elisa Mazzoni, Giuseppe De Rito, Carlo Contini

**Affiliations:** 1 Department of Medical Sciences, Infectious Diseases and Dermatology Section, University of Ferrara, Via Aldo Moro, 44124 Ferrara, Italy; 2 Orthopaedic Ward, Casa di Cura Santa Maria Maddalena, Via Gorizia, Occhiobello, 45030 Rovigo, Italy; 3 Department of Medical Sciences, Centre for Clinical and Epidemiological Research, University of Ferrara, Via Fossato di Mortara, 64/B, 44121 Ferrara, Italy; 4 Department of Medical Sciences, University of Ferrara, Via Fossato di Mortara, 64/B, 44121 Ferrara, Italy; 5 Department of Environmental Sciences and Prevention, University of Ferrara, Via Luigi Borsari 46, 44121 Ferrara, Italy; 6 RDI Srl Cerba HealthCare, Via Del Santo 147, Limena, 35010 Padua, Italy; 7 Department of Translational Medicine, University of Ferrara, Via Luigi Borsari 46, 44121 Ferrara, Italy; 8 Department of Chemical, Pharmaceutical and Agricultural Sciences, University of Ferrara, Via Luigi Borsari 46, 44121 Ferrara, Italy

**Keywords:** *Staphylococcus aureus*, Panton–Valentine leukocidin (PVL), prosthetic joint infection, DAIR, one stage revision, two stage revision, arthroplasty

## Abstract

The aim of this study was to investigate whether the presence of *Staphylococcus aureus* (SA) producing the Panton–Valentine leukocidin (PVL) affects the outcome of Prosthetic Joint Infection (PJI). Patients with acute and chronic PJI sustained by SA were prospectively enrolled at the orthopedic unit of “Casa di Cura Santa Maria Maddalena”, from January 2019 to October 2021. PJI diagnosis was reached according to the diagnostic criteria of the International Consensus Meeting on PJI of Philadelphia. Synovial fluid obtained via joint aspirations was collected in order to isolate SA. The detection of PVL was performed via real-time quantitative PCR (RT-qPCR). The outcome assessment was performed using the criteria of the Delphi-based International Multidisciplinary Consensus. Twelve cases of PJI caused by SA were included. Nine (75%) cases were acute PJI treated using debridement, antibiotic and implant retention (DAIR); the remaining three (25%) were chronic PJI treated using two-stage (n = 2) and one-stage revision (n = 1), respectively. The SA strains that tested positive for PVL genes were 5/12 (41.6%,). Treatment failure was documented in three cases of acute PJI treated using DAIR, all supported by SA–PVL strains (*p* < 0.045). The remaining two cases were chronic PJI treated with a revision arthroplasty (one and two stage, respectively), with a 100% eradication rate in a medium follow-up of 24 months. Although a small case series, our study showed a 100% failure rate in acute PJI, probably caused by SA PVL-producing strains treated conservatively (*p* < 0.04). In this setting, toxin research should guide radical surgical treatment and targeted antibiotic therapy.

## 1. Introduction

The number of total joint arthroplasty (TJA) surgeries being performed has constantly grown in recent years, mainly due to demographic trends, with more elderly people being less inclined to tolerate motor restrictions [1]. This higher request for surgical procedures has led to an increasing amount of patients at risk of postoperative complications. In this regard, periprosthetic joint infection (PJI) has been identified as one of the most damaging healthcare-associated infections (HAI) in orthopedic surgery [2]. The pathogenesis of PJI is related to the growth of pathogens in structures called “biofilms” that hinder diagnosis and make eradication a challenge [3]. Risk factors for PJI are obesity, previous surgery, smoking, and chronic diseases (e.g., rheumatoid arthritis, diabetes mellitus), as well as exogenous factors such as the timing and selection of antibiotic prophylaxis, extended operation times, and blood transfusions [4,5,6,7,8]. Acute PJIs (early or late haematogenic ones) are treated conservatively with debridement, irrigation and implant retention (DAIR) [9]. The success rates of this procedure can reach 80% in the presence of the following selection criteria: (1) the stability of the prosthesis; (2) the absence of a sinus tract; (3) the sensitivity of the pathogen to antibiotics active on the biofilm; and (4) the experience of symptoms for less than 3 weeks [3]. In contrast, in acute PJIs that do not meet the aforementioned criteria, as well as in chronic PJIs, a more radical treatment involving prosthetic implant replacement in one or two stages is recommended [3]. The Staphylococcus genus causes up to two-thirds of PJI [3,10,11,12], with *Staphylococcus aureus* (SA) being the most frequently isolated pathogen, and a significant portion of staphylococcal isolates being methicillin-resistant. Although they have a lower mortality rate than other staphylococcal infections, PJIs caused by SA have a poor prognosis due to the high risk of treatment failure in the medium-long term.

The treatment of staphylococcal PJI requires a multidisciplinary management based on radical surgical cleaning and targeted antibiotic therapy. Despite this combined approach, PJIs sustained by SA are particularly aggressive and often burdened by a negative outcome, characterized by an inclination towards chronicity and relapse [13,14]. The key to the success of SA lies in its production of a broad repertoire of virulence factors in combination with an acquired resistance to antibiotics (e.g., methicillin, fluoroquinolones) and a marked ability to persist within infected osteocytes [15]. Through surface adhesins, it can bind and colonize several surfaces, both organic (e.g., bone) and inert (e.g., medical devices, prosthesis), with a subsequent marked propensity for biofilm formation. Furthermore, SA produces several extracellular virulence factors, such as α-hemolysin, exfoliative toxins, Panton–Valentine leukocidin (PVL) or phenol-soluble modulins. PVL is a two-component pore-forming toxin that causes a cytolytic efflux of molecules and metabolites from cytoplasmic membranes [16], with resultant leukocyte destruction and tissue necrosis [17]. In addition, PVL acts as a powerful polymorphonuclear neutrophil chemotactic agent [18] and has been associated with negative prognostic events such as severity and recurrence [19]. SA–PVL isolates can be found in both methicillin-susceptible SA (MSSA) and methicillin-resistant SA (MRSA) strains [20].

The pathogenetic significance of PVL in skin and soft tissue infections (SSTI) has been established in earlier investigations, indicating a strong correlation between PVL and deep SSTIs [21], which frequently recur if not identified and surgically treated [22]. Other invasive diseases, such as pneumonia, musculoskeletal illness, and bacteremia, are much less common and are only secondarily associated with PVL. In contrast, infection with a PVL-positive strain does not seem to have any bearing on how staphylococcal pneumonia, adult bacteremia, or hidradenitis suppurativa (HS) turn out clinically [21,23]. Over the past ten years, SA PVL-positive strains have been linked to an upsurge in pediatric bone and joint infections [24,25].

In contrast, the prevalence of PJI associated with PVL has been poorly reported and little is known about the outcome of this infection. On this basis, the main endpoint of our study was to investigate whether the presence of SA–PVL strains causing PJI is associated with treatment failure.

The secondary aims were to establish whether the presence of PVL (1) was associated with systemic infections with the isolation of SA from blood cultures and synovial fluid, and (2) affected the kinetics of blood tests (e.g., white blood cells, inflammation markers), thus slowing down their normalization during therapy.

## 2. Material and Methods

### 2.1. Patient Population and Study Design

In the present study, we included all adult subjects affected by acute and chronic PJI sustained by SA (hip, knee, shoulder) that were consecutively referred to the orthopedic unit of “Casa di Cura Santa Maria Maddalena”, a third-level reference center for orthopedic surgery located in Veneto Region (Italy), from January 2019 to October 2021. The diagnosis of PJI was reached according to the diagnostic criteria of the International Consensus Meeting (ICM) on PJI of Philadelphia 2018 (https://icmphilly.com accessed on 1 January 2019). All enrolled patients underwent revision surgery (DAIR, one or two-stage revision) and antibiotic therapy for 12 weeks [26].

The patients’ demographics (age, sex), major comorbidities (obesity was defined as body mass index ≥ 30) and medications, smoking status, drug addiction, American Society of Anesthesiologists (ASA) class [27], length of surgery, total length of hospital stay, history of previous PJI or other staphylococcal infections, infection type, clinical signs at presentation and microbiological documentation, the time frame from arthroplasty implantation to infection onset, type of surgical intervention (DAIR vs non-DAIR procedures), antimicrobial treatment (regimen, duration) and final outcome were collected. Laboratory tests, such as those determining the white blood cell and neutrophils count, inflammation markers (C-reactive protein, CRP) and the erythrocyte sedimentation rate (ESR), had been recorded at onset, upon hospital discharge, and at one, two and three months during antibiotic treatment.

The outcome assessment in terms of treatment success or failure was performed using the criteria of the Delphi-based International Multidisciplinary Consensus [28]. Patients were excluded in the presence of the following: (i) concomitant peri-prosthetic fracture, (ii) frequent prosthetic dislocation, (iii) an intra-articular volume of synovial fluid less than 3 mL, and (iv) the use of systemic antibiotics during the 2 weeks before inclusion. The study was approved by the institutional review board. All the enrolled patients provided their written informed consent before participation. The study was conducted in accordance with the Declaration of Helsinki. No funding from external sources was used to carry out this study. 

### 2.2. Specimen Collection 

Joint aspirations were performed by orthopedic surgeons according to a standardized aseptic technique before incision of the joint capsule in the operating room. In addition, during revision surgeries, a number of 3–5 periprosthetic tissue samples were collected for microbiological and histopathological analysis. 

### 2.3. Determination of Synovial Fluid Leukocyte Count and Percentage Granulocytes

A vial containing ethylenediaminetetraacetic acid (EDTA) was used to collect 1 mL of synovial fluid for the purpose of assessing the leukocyte count and percentage of granulocytes. For 10 min at room temperature, clotted specimens were incubated with 10 μL of hyaluronidase (Sigma-Aldrich Chemie, Taufkirchen, Germany). The test was carried out using an automated hematology analyzer (XE-2100, Sysmex, Norderstedt, Germany) applying flow-cytometry.

### 2.4. Conventional Microbiological Tests

Here, 0.1 mL aliquots of each sample of synovial fluid were added to thioglycolate broth and tryptic soy agar containing 5% sheep blood for aerobic and anaerobic cultures. With the exception of the blood culture bottles, tissue samples were cultured as stated for synovial fluids. For 14 days, all culture mediums were incubated at 35 °C. An automatic bacteriological analyzer, VITEK 2 (bioMérieux, Marcy L’Etoile, France), was used to identify and test the susceptibility of the isolated bacteria.

### 2.5. PVL Assay

Prior to nucleic acid extraction and PCR analysis, the SA culture suspensions were kept at 4 °C. As previously mentioned, bacterial DNA was extracted using the commercial kit RIDA^®^GENE PVL test, and real-time quantitative PCR (RT-qPCR) analysis was performed by amplifying a PVL-specific fragment (Panton–Valentine Leukocidine lukF–PV). [23]. In a tube, *SA* culture suspensions were added with lysis Buffer (200 µL). The preparation consisted of heating, shaking at 95 °C for 10 min in a heating block, centrifuging for 1 min at 12,000 rpm, and applying the supernatant as the sample. Each sample was processed simultaneously with negative controls, represented by samples without DNA (real-time PCR mix), to determine whether cross-contamination had occurred during the DNA extraction, purification, and PCR procedures. Internal Control DNA (ICD), which is part of the RIDA^®^GENE PVL test, served as both an extraction control for the sample preparation process and a PCR inhibition control. The RT-qPCR reaction contained 20 µL of master mix and 5 µL of each sample. The positive control had a concentration of 10^3^ copies/μL. Duplicate replica tests were conducted to analyze samples and controls. Following the manufacturer’s instructions, samples were run through the Biorad CFX96 Touch Real-Time PCR Detection System (Milan, Italy) equipment and analyzed using the Bio-Rad CFX Manager software. The results of both positive and negative controls must be accurate. In each RT-qPCR cycle, 5103 copies were utilized. The RIDA^®^GENE PVL detection limit for the reaction was 5 copies of DNA.

### 2.6. Statistical Analysis 

Variables related to the patients’ characteristics, the type of intervention, and the pre-/post-operative and follow-up laboratory and microbiological tests were tabulated as mean (±standard deviation) or median [interquartile range] in the case of deviation from normal distribution, or as frequencies and percentages in the case of categorical variables. The grouping of cases was carried out via positivity testing for PVL, which is the study exposition. Comparisons between the positive and negative PVL test groups were made using a *t*-test for continuous variables (Wilcoxon test for non-normally distributed variables) and a chi-squared test for categorical variables. Longitudinal data were modeled using two-way analysis of variance without any adjustment for covariates. All statistical tests were two tailed and a significance level of 0.05 was defined for the acceptance of the tested hypothesis, if not specified differently. All analyses were carried out using R version 3.6.2 (R Core Team (2019)).

## 3. Results

### 3.1. Patient Characteristics

Twelve cases of PJI caused by SA were included (Table 1). Acute PJIs were detected in 9 (75%) cases out of 12 and were treated with DAIR, with a mean time of onset of 314.5 days (range 7–1826). Among these, three (30.0%) cases were hematogenous PJIs. The remaining three cases (25%) were chronic PJIs treated with two-stage revision (n = 2) and one-stage revision (n = 1), respectively. Among the latter, the infection was diagnosed with a medium time of 702.25 days after primary arthroplasty (range 70–1826).

The median age was 65 years (range 43–87), and half of the enrolled patients were female. The principal comorbidities were obesity (n = 12) and rheumatologic disorders (n = 11), while diabetes mellitus was detected in only one case; smoker status and drug addiction were found in three and two patients (25% and 16.67%, respectively). In six (50%) cases, a previous or concurrent SA infection was recorded: skin and soft tissue (SSI) (n = 1), spondylodiscitis (n = 1), previous SA PJI (n = 2) and septic arthritis (n = 1), and endocarditis of native valve (n = 1). None of the collected PJIs were polymicrobial. Among the 12 isolated SA, only 2 (16.67%) were methicillin-resistant *S*. *aureus.*

In all primary arthroplasty procedures, prophylaxis had been performed with cefazolin. In the four cases with a previous history of staphylococcal infection, vancomycin or a combination of cefazolin + vancomycin was administered. The correct dosage and timing of antimicrobial prophylaxis were ensured in all the analyzed cases (Table 2).

### 3.2. Microbiological Assessments

SA strains tested positive for PVL genes in 5 out of 12 cases (41.6%). Of these, two strains were MR. Three of the PVL-positive cases were in the DAIR group, and two underwent revision (one and two stage, respectively). Table 1 reports the main PJI patients’ characteristics according to their positivity for PVL genes. Among the PVL-negative patients, 14.3% had diabetes (1/7) and took anticoagulant drugs (Warfarin, 1/7). On the other hand, among the PVL-positive patients, 40% presented injection drug abuse (2/5), while 20% showed HCV/HBV active infection (1/5). Overall, the studied patients did not present any rheumatological disorders, without treatment with monoclonal antibodies or corticosteroids.

In Table 2, the main PJI patients’ procedural data according to their positivity for the PVL gene are reported.

One third of patients experienced systemic symptoms (e.g., fever) besides edema, hyperemia, and functional impotence at the surgical site. In these cases (n = 4), the isolation of SA from blood culture, in addition to synovial fluid, was performed. Severe invasive disease requiring Intensive Care Unit (ICU) admission was not registered in any of the analyzed cases, although the occurrence of SA-supported bacteremia required longer hospitalization (*p* < 0.028). None of the four SA-invasive infections were sustained by a PVL-producing strain, as well as none of the hematogenous PJIs.

No significant differences were found in the hematochemical parameters considered at PJI onset (Table S1) and at discharge (Table S2).

**Figure 1:**
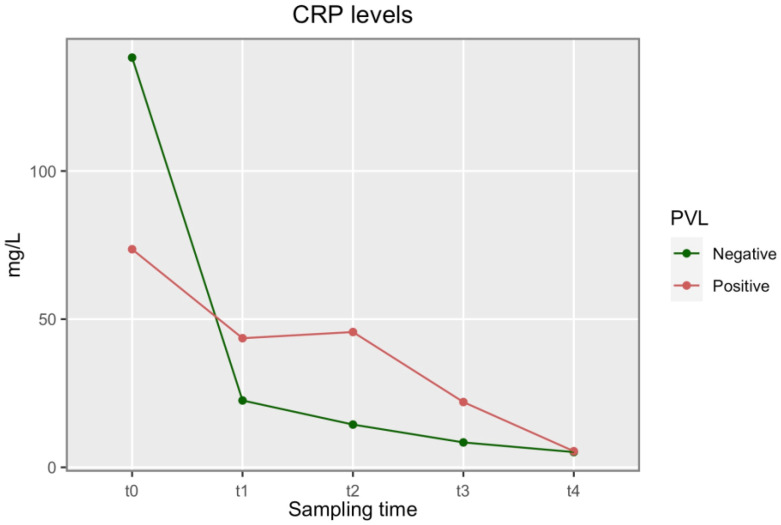
CRP levels at pre-operative (t0), discharge (t1) and during antibiotic treatment at 1 (t2), 2 (t3) and 3 (t4) months. A difference between the two groups of patients was observed in the average CRP values across the sampling time, *p* < 0.05.

On the contrary, a different kinetic of CRP was recorded during the planned twelve weeks of antibiotic therapy, with a slower decline in the PVL-positive group (*p* = 0.03; Figure 1).

### 3.3. Clinical Outcomes and Microbiological Associations

Treatment failure was documented in the three cases of acute PJI (hip = 1, knee = 1, shoulder = 1) caused by SA–PVL strains and treated with DAIR (*p* < 0.045). Failures occurred before the end of the planned 12 weeks of antibiotic therapy and required the removal of the prosthetic hardware.

The remaining two cases of PJI caused by the SA PVL-producing strains were chronic PJIs treated with a revision arthroplasty (one and two stage, respectively) and targeted antibiotic therapy for 12 weeks; these resulted in a 100% eradication rate in a medium follow-up of 24 months (range 15–33). The surgical treatment, antibiotic regimens, and outcome of the total PJI cases are shown in Table S3.

**Figure 2:**
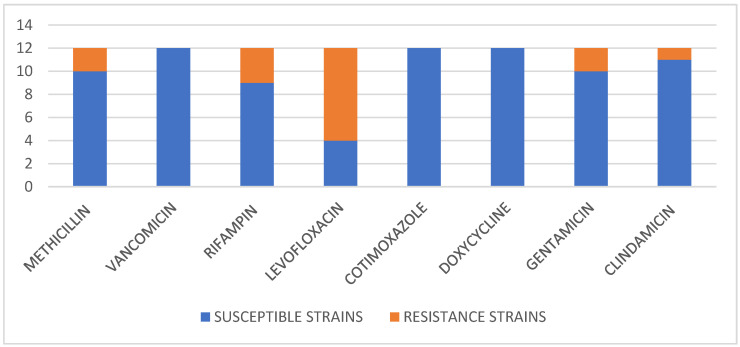
Resistance to/susceptibility patterns of the most common anti-staphylococcal antibiotics detected in the isolated SA strains causing PJI.

The antimicrobial resistance profile of all SA isolates is reported in Figure 2.

The three PVL–SA strains that failed with conservative treatment were all sensitive to methicillin, fluoroquinolone, cotrimoxazole and doxycycline. Resistance to rifampicin was found in only one out of three cases (33.3%). With regard to the two cases of PJI caused by PVL–SA strains and treated with revision arthroplasties, resistance to fluoroquinolone was detected in both cases (100%), while resistance to methicillin was detected in only one (50%). None of the patients affected by a SA PVL-producing strain had a multifocal infection.

## 4. Discussion

SA continues to be one of the most challenging microorganisms in human diseases. It is a commensal that colonizes the skin, but can also become a pathogen commonly causing PJI [10].

Previous works have indicated that the male sex and comorbidities such as smoking, obesity and diabetes are risk factors for osteoarticular infections caused by SA PVL-producing strains [29]. In the present study, the lack of a statistically significant difference in the comparison of risk factors between the two groups could be due to the fact that the main risk factors for PVL match those for SA–PJI. However, host-related factors seemed to play a predominant role in favoring SA–PJI, since exogenous factors, particularly antimicrobial prophylaxis and operation time, appeared to be well controlled in all analyzed cases. Furthermore, as previously described, patient characteristics can influence the treatment outcome [30,31]. It is therefore not surprising that a higher ASA score, obesity and smoking were represented in two out of three PJI cases that saw failed microbiological eradication. The failed cases involved acute infections (early post-operative) sustained by SA PVL-producing strains, treated with DAIR [9] (*p* < 0.045). The impact of risk factors on the outcome of PJI treated conservatively has not been fully explored so far, but a recent report has proposed the KLIC score (Kidney, Liver Index surgery, Cemented prosthesis, and C-reactive protein) as a risk stratification tool [30]. However, the KLIC score, when used to stratify the risk of relapse in patients undergoing DAIR for acute SA–PJI, in our experience, has shown poor accuracy for outcome prediction since all the three patients who failed microbiological eradication were considered at “low risk” (KLIC score < 4). According to our previous data, the main limit of the score is represented by the lack of a validated microbiologic parameter [32].

How long DAIR remains a useful option after PJI presentation is still a matter of discussion [33,34]. The greatest chance of success is reserved for infections whose symptoms have been present for less than three weeks, since a mature biofilm has not yet been formed. However, in our case series, although this time interval was strictly observed, microbiological eradication could not be achieved in three cases, due to an early infection relapse that occurred before the completion of the planned 12 weeks of therapy. Furthermore, to increase the success of DAIR, antibiotic therapy that is highly active against SA biofilm should be administered, specifically rifampin in combination with another anti-SA drug, and not an active biofilm (e.g., levofloxacin, cotrimoxazole, doxycycline) [35]. By analyzing the antimicrobial resistance patterns of the failed cases, however, it is surprising to note that treatment failure also occurred in two PVL-producing SA–PJI, despite a proved sensitivity to rifampin and the administration of a combination therapy (rifampin + not-biofilm active antibiotic).

Over the last decade, the emergence of PVL-producing SA strains has become an increasing concern due to their ability to induce severe osteoarticular and multifocal infections, particularly in children [36,37,38]. Moreover, a new species of particularly aggressive community-acquired MRSA, producing PVL, has been reported to be responsible for deep, multifocal skin abscesses in long-term familial clusters [39]. Invasive disease may occur as a complication of a preceding SSTI or a musculoskeletal infection, and often requires admission to an intensive care unit (ICU) [35].

In contrast, data on the severity of prosthetic infections sustained by SA PVL-positive strains are lacking and little is known about their outcome.

Our study showed a 100% failure rate in acute PJI caused by SA PVL-producing strains treated conservatively (*p* < 0.04). This finding appears to strongly discourage the application of DAIR in these cases, regardless of rifampin susceptibility. Since eradication in the current study was only possible with implant removal, a two-stage exchange should be used as the primary intervention if the PJI is caused by a PVL-producing strain.

PVL production, on the other hand, has not been associated with an increased risk of invasive infection or bacteremia during PJI, in contrast to other reports in the literature [36,38].

Furthermore, no statistically significant difference was found between the two groups in the kinetics of the hematochemical tests at PJI onset and at hospital discharge (Table S1). This finding also contrasts with other reports in the literature, where the presence of PVL is associated with invasive infections and consequently with systemic inflammation. However, the statistically significant difference found in the drop in CRP during antimicrobial therapy between the two groups (*p* = 0.03; Figure 1) seems to suggest that PVL production in the context of PJI correlates with a marked local aggressiveness.

This study is not free of limitations. First, the formation of biofilms by infecting strains has not been explored. Second, the relatively small number of patients enrolled in this study is due to the low frequency of this complication in arthroplasty. The baseline characteristics of the patients show no significant differences between PVL that tested positive and negative, so they are therefore comparable. Nevertheless, it is worth noting that the small cohort does not provide an adequate sample for statistical inference and more sophisticated statistical modeling procedures for causality testing is needed. Furthermore, no causal inference was carried out to enforce the experimental evidence and to control for confounders. Thus, these preliminary findings must be validated in a wider cohort of patients with the same methodological approach.

## 5. Conclusions

Acute PJI caused by SA–PVL strains in our cases seems to be burdened by the failure of conservative treatment rather than invasive infection. In these cases, toxin research should guide the choice of treatment. Positivity for PVL-encoding genes should require multidisciplinary management, including radical surgical debridement with two-stage exchange and targeted antibiotic therapy. Further studies are needed in order to confirm our preliminary results.

## Figures and Tables

**Table 1:** Patient characteristics at baseline.

	PVL Negative	PVL Positive	Overall	*p*
Patients Characteristics	n = 7	n = 5	n = 12	
Gender Male, n (%)	3 (42.9)	3 (60.0)	6 (50.0)	0.56
Age (years), mean (SD)	65.71 (7.85)	64.20 (18.65)	65.08 (12.67)	0.85
BMI ^a^, mean (SD)	32.92 (7.67)	32.06 (6.49)	32.56 (6.90)	0.84
Active smoker, n (%)	2 (28.6)	1 (20.0)	3 (25.0)	1.00
Diabetes	1 (14.3)	0 (0.0)	1(8.3)	0.38
Anticoagulants, n (%)				0.22
Cardioaspirin	5 (71.4)	2 (40.0)	7 (58.3)	
VKA ^h^	1 (14.3)	0 (0.0)	1 (8.3)	
DAOA ^i^	0 (0.0)	0 (0.0)	0 (0.0)	
ASA ^b^ score, n (%)				0.52
2	1 (14.3)	2 (40.0)	3 (25.0)	
3	6 (85.7)	3 (60.0)	9 (75.0)	
Previous or concomitant SA ^c^ infections, n (%)				1.00
SA–PJI ^d^ (hip) five years earlier	0 (0.0)	1 (20.0)	1 (8.3)	
SA–septic arthritis (shoulder) one year earlier	0 (0.0)	1 (20.0)	1 (8.3)	
Concomitant SA–endocarditis	1 (14.3)	0 (0.0)	1 (8.3)	
SA–PJI (knee) one year earlier	1 (14.3)	0 (0.0)	1 (8.3)	
Concomitant SA–spondylodiscitis	1 (14.3)	0 (0.0)	1 (8.3)	
Concomitant SA–cellulitis	1 (14.3)	0 (0.0)	1 (8.3)	

^a^: body mass index; ^b^: American Society of Anesthesiologists class; ^c^: *Staphylococcus aureus*; ^d^: prosthetic joint infection. ^h^: vitamin K anticoagulant therapy; ^i^: direct-acting oral anticoagulant therapy.

**Table 2:** Prophylaxis and procedural data of treated patients.

	PVL Negative	PVL Positive	Overall	*p*
Prophylaxis/Procedure	n = 7	n = 5	n = 12	
Antimicrobial prophylaxis at first implant, n (%)				0.82
cefazolin 2 g	5 (71.4)	3 (60.0)	8 (66.7)	
cefazolin 2 g, vancomycin 1.5 g	0 (0.0)	1 (20.0)	1 (8.3)	
vancomycin 1 g	1 (14.3)	1 (20.0)	2 (16.7)	
vancomycin 1.5 g	1 (14.3)	0 (0.0)	1 (8.3)	
Length of surgery (first implant > 120 m), n (%)	3 (42.9)	2 (0.40)	5 (41.67)	0.20
PJI ^d^ anatomical location, n (%)				0.38
Hip	2 (28.6)	2 (40.0)	4 (33.3)	
Knee	5 (71.4)	2 (40.0)	7 (58.3)	
Shoulder	0 (0.0)	1 (20.0)	1 (8.3)	
PJI onset time (weeks), median [IQR]	5.00 [3.50, 11.00]	130.00 [4.00, 156.00]	7.50 [3.75, 136.50]	0.57
Surgical procedure type, n (%)				0.31
DAIR ^e^	6 (85.7)	3 (60.0)	8 (75.0)	
Revision arthroplasty (one or two stage)	1 (14.3)	2 (40.0)	3 (25.0)	
Fever (>38 °C) at onset, n (%)	4 (57.1)	0 (0.0)	4 (33.3)	0.08
Sinovial fluid cultures, n (%)				0.79
MRSA ^f^	1 (14.29)	1 (20.0)	2 (16.7)	
MSSA ^g^	6 (85.71)	4 (80.0)	9 (83.33)	
Blood cultures positivity, n (%)	4 (57.1)	0 (0.0)	4 (33.3)	0.15

^d^: prosthetic joint infection; ^e^: debridement, antibiotic, implant retention; ^f^: methicillin-resistant *Staphylococcus aureus*; ^g^: methicillin-sensitive *Staphylococcus aureus.*

## Data Availability

All data generated or analyzed during this study are included in this manuscript. Detailed data are available from the corresponding author upon request.

## Supplementary Materials

The following supporting information can be downloaded at: https://www.mdpi.com/article/10.3390/biomedicines11061767/s1, Table S1: Hematochemical parameters at PJI onset; Table S2: Hematochemical parameters and length of hospitalization at discharge (after IV antibiotic treatment); Table S3: Microbiological data, antimicrobial therapy, type of surgical treatment and outcome of patients with PJI caused by Panton–Valentine (PVL)-producing *S. aureus*.
