# Supplementary material for: Acute Prosthetic Joint Infections with Poor Outcome Caused by Staphylococcus Aureus Strains Producing the Panton–Valentine Leukocidin
Source: Biomedicines. 2023 Jun 20;11(6):1767. doi: 10.3390/biomedicines11061767 (PMC10295985; doi:10.3390/biomedicines11061767)
Supplement: Supplementary file 1 [file biomedicines-11-01767-s001.zip › biomedicines-2426040-supplementary.pdf]

Supplementary materials

Table S1. Haematochemical parameters at PJI onset.

	PVL negative	PVL positive	Overall	p
N	(n=7)	(n=5)	(n=12)	
CRP[h], median [IQR]	86.00 [75.15, 195.60]	57.00 [38.00, 99.00]	83.50 [64.88, 150.30]	0.12
ESR[i], mean (SD)	80.71 (16.87)	77.60 (14.21)	79.42 (15.20)	0.74
WBC[l], mean (SD)	9118.24 (7043.68)	9078.21 (5116.14)	9101.56 (6048.17)	0.99
Neutrophils, mean (SD)	7375.90 (6198.02)	8061.12 (4580.38)	7661.41 (5357.93)	0.84
HB[m], mean (SD)	11.30 (1.50)	12.56 (1.59)	11.82 (1.60)	0.19
Proteins, mean (SD)	6.15 (0.77)	6.00 (0.78)	6.08 (0.72)	0.79

[h]: C reactive protein; [i]: erythrocyte sedimentation rate; [l]: white blood cells; [m]: haemoglobin.

Table S2. Haematochemical parameters and length of hospitalization at discharge (after iv antibiotic treatment).

	PVL negative	PVL positive	Overall	p
N	(n=7)	(n=5)	(n=12)	
CRP[h], median [IQR]	20.80 [13.54, 23.12]	27.00 [16.50, 81.00]	21.70 [14.28, 34.50]	0.37
ESRi, median [IQR]	42.00 [38.50, 56.00]	48.00 [45.00, 53.00]	46.50 [39.25, 55.75]	0.52
WBC[l], mean (SD)	6697.14 (1848.65)	8738.00 (2179.68)	7547.50 (2167.06)	0.11
Neutrophils, mean (SD)	3611.43 (1387.04)	5320.00 (2715.17)	4323.33 (2122.31)	0.18
HB[m,] mean (SD)	11.39 (1.04)	10.88 (1.30)	11.18 (1.13)	0.47
Proteins, mean (SD)	5.67 (0.91)	5.58 (0.73)	5.61 (0.74)	0.89
Hospitalizatioz (days), median [IQR]	25.00 [17.00, 40.50]	14.00 [10.00, 15.00]	15.50 [14.00,26.75]	0.03

[h]: C reactive protein; [i]: erythrocyte sedimentation rate; [l]: white blood cells; [m]: haemoglobin.

Table S3. Microbiological data, antimicrobial therapy, type of surgical treatment and outcome of patients with PJI due to Panton Valentine (PVL)-producing *S. aureus*.

N.	Sex	Age	Methicillin susceptible/ resistance SA strain	PVL	SURGICAL PROCEDURE	I.V.[n] ANTIBIOTIC (days of treatment)	SEQUENTIAL ORAL ANTIBIOTIC (days of treatment)	OUTCOME (n. of days)*
1	F	60	MSSA[g]	-	DAIR[d]	vancomycin 2 g (14)	cotrimoxazole 3 x 160/800 mg + rifampin 900 mg (70)	REMISSION (730)
2	F	79	MSSA	+	DAIR	vancomycin 2 g (15)	cotrimoxazole 3 x 160/800 mg (63)	FAILURE (63)
3	M	46	MRSA[f]	+	TWO-STAGE REVISON	vancomycin 2 g (10)	Doxycycline 2 x 100 mg (77)	REMISSION (730)
4	M	59	MRSA	-	DAIR	daptomicin 850 mg (49)	Doxycycline 2 x 100 mg (35)	REMISSION (730)
5	F	79	MSSA	-	TWO-STAGE REVISON	vancomycin 2 g (28)	Doxycycline 2 x 100 mg (63)	REMISSION (365)
6	F	74	MSSA	-	DAIR	vancomycin 2 g (9), cefazolin 3 x 2 g (6)	Cotrimoxazole 3 x 160/800 mg + rifampin 900 mg (70)	REMISSION (545)
7	M	87	MSSA	+	ONE-STAGE REVISION	vancomycin 2 g (7), cefazolin 3 x 2 g (7)	Doxycycline 2 x 100 mg (70)	REMISSION (425)
8	M	43	MSSA	+	DAIR	vancomycin 2 g (8), cefazolin 3 x 2 g (8)	Levofloxacin 2 x 500 mg + rifampicin 900 mg (21)	FAILURE (37)
9	M	61	MSSA	-	DAIR	vancomycin 2 g (9), cefazolin 3 x 2 g (10)	Levofloxacin 2 x 500 mg +rifampicin 900 mg (70)	REMISSION (425)
10	F	69	MSSA	-	DAIR	vancomycin 2 g (12), cefazolin 3 x 2 g (13)	Cotrimoxazole 3 x 160/800 mg + rifampin 900 mg (63)	REMISSION (365)
11	M	64	MSSA	-	DAIR	vancomycin 2 g (56) + rifampin 900 mg (42)	Cotrimoxazole 3 x 160/800 mg + rifampin 900 mg (42)	REMISSION (665)
12	F	68	MSSA	+	DAIR	daptomicin 700 mg (7)	Doxycycline 2 x 100 mg (70)	FAILURE (77)

[n]: Intravenous; [g]: meticillin-sensitive *Staphylococcus aureus*; [f]: meticillin-resistant *Staphylococcus aureus;* [d]: debridment, antibiotic, implant retention. *Number of days: (1) after open debridement in case of failure and (2) after finishing antibiotic in case of remission.
